# Digital delivery of the GDS method for chronic lumbosacral discogenic pain: A pilot study aligning Sustainable development Goals three and four

**DOI:** 10.12669/pjms.42.9.16255

**Published:** 2026-09

**Authors:** Haroon Mansha, Saleh Shah, Muhammad Naveed Babur, Muhammad Junaid Ijaz

**Affiliations:** 1 Dr. Haroon Mansha, Faculty of Allied Health Sciences, Superior University, Lahore, Pakistan; 2 Dr. Saleh Shah, PhD Health Management. Faculty of Allied Health Sciences, Superior University, Lahore, Pakistan; 3 Muhammad Naveed Babur, PhD Rehabilitation Sciences, Faculty of Allied Health Sciences, Superior University, Lahore, Pakistan; 4 Muhammad Junaid Ijaz, PhD Rehabilitation, Faculty of Allied Health Sciences, Superior University, Lahore, Pakistan

**Keywords:** Chronic Back Pain, Lumbosacral Region, Pilot Study, Quality of Life, Smartphone App

## Abstract

**Background and Objectives:**

A person’s quality of life and everyday functioning may be significantly restricted by chronic back pain including lumbosacral discogenic pain. The purpose of this study is to evaluate the GDS method’s initial effects on patients with chronic lumbosacral discogenic pain in terms of both pain and quality of life delivered through a smart phone application in a pilot study.

**Methodology:**

Between July 2025 and September 2025, a one-group pretest-posttest design was chosen, and a total of fifteen patients (six male and nine female; mean age 45± 9.0 years; range, 35 to 65 years) were enrolled in the study. Study was Designed at Superior University, Lahore and conducted at Seyal Medical Center. A free local designed smartphone application was used to deliver the 6-week GDS program. Daily reminders, video instructions, and guided postural exercises were all part of the intervention aligning it with SDG three and four. The SF-36 Quality of Life Survey and the ODI scale for pain were used as outcome measures both before and after the intervention. Data on adherence and user engagement were also gathered.

**Results:**

From pre-intervention to post-intervention, all SF-36 short form domains showed a statistically significant increase (p <.05). A small scale change (40%) was seen in the ODI scale after the intervention. Eighty percent of participants completed at least five sessions per week on average.

**Conclusion:**

Small percentage of patients with chronic discogenic pain showed improvements in pain intensity, functional disability, and certain quality-of-life domains after completing a six-week digitally delivered GDS program. Platforms for digital physiotherapy need to define more parameters, so it could provide a scalable and affordable addition to conventional care.

## INTRODUCTION

One of the main causes of disability in the world is still chronic low back pain.^1^ By the early 2026, the global health impact is escalating, primarily as a result of demographic shifts toward an older population and the prevalence of metabolic risk factors like physical inactivity and high BMI.^1^,^2^ Management protocols prioritize non-pharmacological interventions, emphasizing patient education and maintaining physical activity as first-line treatments. Secondary options include the short-term use of NSAIDs or manual therapies like spinal manipulation and acupuncture to facilitate a return to daily function.^2^ Surgical and interventional procedures are strictly reserved for severe structural pathologies or progressive neurological deficits that fail to respond to conservative care. Discogenic pain, which results from degenerative or structural alterations in the intervertebral disc, accounts for up to 40% of chronic cases. Unlike radicular discomfort, discogenic pain is typically deep, axial, and exacerbated by prolonged postures such as sitting or lifting. Diagnosis is also very mixed by performing multiple manual tests and not relying on a single one. The best diagnosis comes with the gold standard MRI, which confirms the discogenic involvement. Many treatments options are also being practiced prioritizing the conservative management.^3^ Those patients, who never choose surgery as a recommended option and remain in episodic back pains in the later life, are always in need of the best conservative management for the improvement in quality of life.^4^ The Godelieve Denys-Struyf (GDS) technique is a physio-therapeutic method based on the concept of physiological muscle chains, which integrate biomechanical and psychomotor components. The approach claims that persistent pain is often caused by instability or rigidity within specific muscle chains, such as the Posterior-Median (PM), Antero-Median (AM), or other chains in lumbar pathology. Traditional GDS therapy has theoretical and clinical benefits, but accessibility is limited by the need for repeated in-person sessions and specialized practitioner training.^5^

By facilitating remote, organized, video-guided interventions, digital delivery may offer a potential remedy. Digital health platforms deliver cost-effective, accessible care via smartphone apps, reducing the necessity of clinic visits for patients seeking non-surgical solutions for chronic LBP.^6^

The rationale of this study was to determine if patients with persistent lumbosacral discogenic pain could benefit from a digitally delivered GDS program in terms of pain intensity, functional impairment, and quality of life.

Given the limited size of this treatment-resistant clinical pool, the sample was scoped as sufficient for within-subject pre–post comparison rather than population-level inference and feasibility data to inform a future, adequately powered study.

## METHODOLOGY

A one-group, prospective, longitudinal pretest-posttest design was used. Between July 2025 and September 2025, Fifteen patients were included and the study was performed at Seyal Medical Center in Pakistan

### Ethical Approval:

Study was designed and approved at Superior University, Lahore with IRB/FAHS/REHAB/06/11/PhD/RS-4629, dated April 17, 2025 and Registered with Clinical Trial number NCT07068321. ***Inclusion criteria:***

- Patients with MRI-confirmed disc degeneration,
- Smartphone ownership with internet connectivity, digital literacy, and chronic lumbosacral discogenic pain (>3 months) between the ages of 35 and 65 were included in the study.

### Exclusion criteria:

- Prior spinal surgery,
- Pregnancy,
- Serious mental disease,
- Osteoarthritis grade 3 & 4,
- Vertebral fractures, and cancers.

A free local android application was shared by the therapist and installing the apk file (Physio for back pain) at the time of patient consent. The app was not launched commercially and limited it to this pilot study patients only, later be available at android and apple stores for large scale study. All Patients were educated about the role of digitalization in health care and understanding and promoting smart applications in healthcare knowledge delivery and applicability aligning the SDG 3.4, 3.8 and 4.7, app offered daily 10-minute video guided sessions, educational material with daily reminders. App started with a one-time baseline data collection questionnaire with built in SF-36 short form and ODI scale, after submitting the data the app continues daily GDS exercises which repeated for six weeks. To ensure adherence to the digital delivery, three notifications were enforced to notify patient session automatically when connected to internet if they missed a session, the app locked that day session and moved to next day tasks. After the completion of 6 week time, the app again showed the post data collection questionnaire with built in SF-36 short form and ODI scale along with usability experience data.

The Oswestry Disability Index (ODI) and the SF-36 short form Health Survey were the primary outcomes.^7^,^8^ The app itself calculated the adherence rate, which was the percentage of completed sessions. Paired t-tests were used to compare baseline and post-intervention results. The threshold for statistical significance was fixed at p < 0.05.

## RESULTS

Nine women (60%) and six men (40%) finished the study. The average age was 45 ± 9.0 years. Baseline was collected right after the app installation and before the start of treatment while post data was collected after six weeks. This was all done through the app. All patients data was collected through one click from the app admin panel. All Patients completed the study but with missed session days reducing the adherence rate to the six week plan. The effect of the intervention on patients’ health-related quality of life, as determined by the SF-36 Short Form Health Survey (N=15), was assessed using a paired-samples t-test, shown in Table-I.

**Table-I:** Showing results of SF-36 Short Form Health Survey

	Pre Mean±SD	Post Mean±SD	t-value	P Value
Physical functioning	59.6±13.9	72.4±12.5	4.82	.001
Role limitations due to physical health	56.6±12.4	70.1±11.8	5.11	.001
Role limitations due to emotional problems	60.1±12.4	71.5±10.2	3.78	.002
Energy/fatigue	63.2±14.4	75.8±13.1	4.15	.001
Emotional well-being	62.7±9.3	74.2±8.5	4.63	.001
Social functioning	61.2±10.2	72.9±9.6	4.28	.001
Pain	60.7±11.4	73.5±10.1	4.95	.001
General health	61.6±10.3	70.8±9.8	3.42	.004

Physical Functioning scores increased statistically significantly (p <.001) between pre- and post-intervention. Role Limitations owing to Physical Health also showed substantial improvements (p <.001). Additionally, there was a significant decrease in interference in the Bodily Pain domain (p <.001).

Consistent improvements were seen in the psychological dimensions. There was a significant improvement in emotional well-being (p <.001). Additionally, there was a significant gain in social functioning (p <.001). Although the mean rise in general health views was the lowest, the shift was nonetheless statistically significant (p <.004).

With a mean score of 43.3% at baseline, the majority of the cohort (n=10) was categorized as having Severe Disability based on ODI criteria as shown in Table-II. ODI respond with a small change happened after the intervention. Categorical shifts revealed that six out of 10 participants (60%) in the baseline Severe group demonstrated sufficient functional improvement to transition into the Moderate Disability group post-intervention. The remaining four participants (40%) in the Severe group maintained their severe impairment classification. None of the five participants in the baseline Moderate group shifted categories. Consequently, post-intervention classifications comprised 11 participants (73.3%) with Moderate Disability and four participants (26.7%) with Severe Disability. At the end of the trial, the group mean fell to only 35.9%. Even in cases where complete recovery was not attained, this change indicates a more focus on the patients to be excluded with co-morbidities and advanced age group. Patients with digital illiteracy are also a barrier to the digital health delivery and compliance tendency toward functional independence.

**Table-II:** showing results of ODI scale

Disability Category	Score Range	Pre-Intervention (n)	Post-Intervention (n)
Minimal Disability	0% – 20%	0	0
Moderate Disability	21% – 40%	5	11
Severe Disability	41% – 60%	10	4
Crippled	61% – 80%	0	0
Bed-bound/ Exaggerating	81% – 100%	0	0

Mean adherence was 80% of participants completing at least five sessions weekly as shown in Fig.1. Ninety percent expressed satisfaction and willingness to continue such treatments and promoting smart applications in healthcare education, digital delivery and applicability.

**Fig.1:**
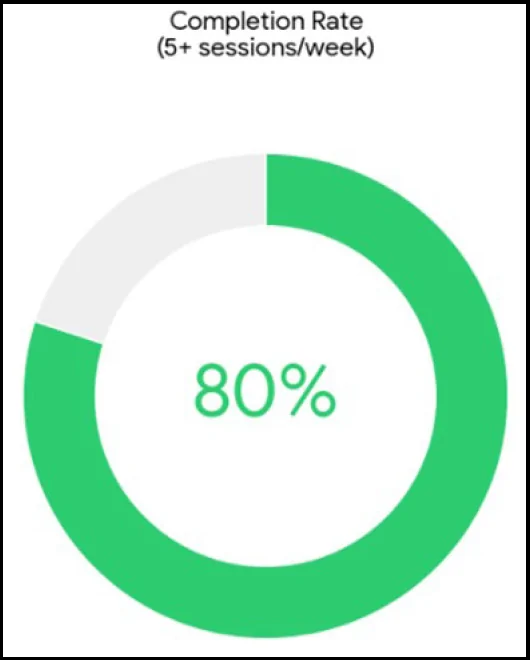
Showing mean adherence rate.

## DISCUSSION

Total 40% of participants achieved categorical disability improvement while 60% remained in their baseline bracket which highlights key prognostic factors in Digital-delivered exercise therapy. Our exploratory findings suggest that younger age and shorter symptom duration positively influenced intervention success. Older patients or those with long-standing structural disc degeneration may require individualized, hands-on GDS manual therapy alongside digital guidance to achieve clinically meaningful threshold shifts in outcome scales for improvements in pain and disability which is a fastest growing chronic musculoskeletal problem now a days.^9^,^10^ Future fully powered trials should stratify outcomes by age brackets and chronicity to establish targeted clinical profiles for digital rehabilitation.

Similarly, many Studies use ODI as a good tool to assess the severity of chronic back pain with the treatments currently available and for the new treatments being developed to evaluate the quality of life for such kind of patients.^11^,^12^ The relatively adherence rate suggests that digital platforms may enhance accessibility and engagement, particularly when incorporating reminders and replay-able instructional content. Digital platforms and social media are a good source of information including smartphone applications, websites, social media and YouTube videos.^13^ Many patients across globe find such apps cost effective due to more focused, detailed review and multiple treatment guidance aligning the SDG 3.4, 3.8 and 4.7.^14^

The findings of this study suggest that the intervention facilitated a positive shift in the participants’ health-related quality of life. The significant improvements observed in all domains indicate that the intervention was particularly effective at addressing the functional mechanics of daily life, likely by reducing the perceived burden of physical tasks. Lombardo et al. (2023) showed significant improvements in disability scores, slight reduction in leg pain intensity and ODI improvement of 8.7 points in GDS group (p < 0.05).^15^ Our study results showed a very small shift in ODI scores with improvement in quality of life. Marked improvement in Emotional Well-being suggests a psychological benefit that may stem from increased self-efficacy or the therapeutic nature of the intervention itself. Grotle M et al. (2022) showed feasible results in ODI scores of patients with chronic low back pain and disc degeneration delivered via GDS method.^16^ Our study showed only 40% change in ODI Scores in relevant to that study. In another study by Arribas et al. (2015) provided significant evidence through a major cluster-randomized controlled trial where VAS and ODI scores improved as our study results.^17^ A study by Puppin et al. (2011) worked significant on GDS stretching exercises to promote quality of life and reducing functional disability and pain in patients with nonspecific low back pain.^18^ Jesus et al. (2024) evaluated the effect of GDS postural patterns on pain and depression in postpartum women.^19^ Most of the previous studies recommend for a larger scale with large sample size studies for more valid applicability of GDS method treatments for chronic LBP patients.^20^ This pattern often occurs in short-term pilot studies where physical structures or systemic health perceptions require more time to show robust statistical change. Our Study results with not much effective ODI scores suggest that a small sample size pilot study cannot suggest a treatment to be highly effective with digital delivery. These results provide a promising baseline, suggesting that while the intervention is show small effectiveness, a larger sample size or a longer follow-up period with age control and digital literacy might be necessary to capture the full extent of its impact across all dimensions of health.

Godelieve Denis-Struyf (GDS) method has an established body of evidence supporting its efficacy in clinic-based physical therapy; its translation into digital delivery formats has remained largely unstudied. By bridging specialized movement therapy with modern mobile health technology, this study demonstrates that core GDS exercises can be adapted into standard digital protocols for remote management of chronic lumbosacral discogenic pain if larger-longterm scale Randomized trial with subgroup stratification could be added.

### Strengths

It represents one of the preliminary attempts to digitize and operationalize the GDS method via a mobile application, Baseline and post data was collected electronically from the app. Adherence was 80 percent showing interest in digitalization of physiotherapy exercise plans and allowing patients to receive real time adherence to the plan that has to be completed.

### Limitations

The study has a limited sample size, no control group, old age patients, and a brief follow-up period of six months. To verify long-term efficacy, randomized controlled trials with prolonged follow-up are required.

## CONCLUSION

A small percentage of participants achieved categorical disability improvement while 60% remained in their baseline bracket of ODI scale, highlights key prognostic factors in Digital-delivered exercise therapy.

SF 36 short form showed improved quality of life dimensions after completing a six weeks digitally delivered GDS program. Platforms for digital physiotherapy could provide a scalable and affordable addition to conventional care by including more focused inclusion criteria, large scale sampling, randomized controlled trial, age bracket and digital literacy to align the SDG 3.4, 3.8 and 4.7.
